# Platelet Distribution Width Enhances Prediction of Residual Coronary Complexity Beyond Clinical Presentation in Patients Undergoing Culprit-Only PCI

**DOI:** 10.3390/medicina62050864

**Published:** 2026-04-30

**Authors:** Mert Deniz Savcilioglu, Nil Savcilioglu, Kemal Ozan Lule, Emre Atessonmez

**Affiliations:** 1 Cardiology Department, Faculty of Medicine, Gaziantep University, Gaziantep 27410, Turkey; ncelikkalkan@gmail.com (N.S.); emreatessonmez@gmail.com (E.A.); 2 Internal Medicine Department, Faculty of Medicine, Gaziantep University, Gaziantep 27410, Turkey; drkemalozanlule@gmail.com

**Keywords:** platelet distribution width, residual SYNTAX score, multivessel coronary artery disease, percutaneous coronary intervention, risk stratification

## Abstract

*Background and Objectives:* Residual coronary anatomical complexity following culprit-lesion-only percutaneous coronary intervention (PCI) remains a major determinant of clinical outcomes in patients with multivessel coronary artery disease (CAD). Platelet distribution width (PDW), a marker of platelet heterogeneity and activation, has been associated with adverse cardiovascular outcomes; however, its relationship with post-procedural residual disease burden remains unclear. This study aimed to evaluate the association between PDW and residual SYNTAX (Synergy between Percutaneous Coronary Intervention with Taxus and Cardiac Surgery) score and to determine its incremental predictive value beyond established clinical variables. *Materials and Methods:* In this retrospective, single-center study, 140 patients with multivessel CAD undergoing culprit-lesion-only PCI followed by planned staged revascularization were included. Clinical presentation was categorized as chronic coronary syndrome (CCS), non-ST-elevation myocardial infarction (NSTEMI), or ST-elevation myocardial infarction (STEMI). Residual SYNTAX score was calculated after the index procedure, and patients were stratified into low (≤22) and high (≥23) groups. Associations between PDW and residual SYNTAX score were assessed using correlation and regression analyses. Model discrimination and incremental predictive value were evaluated using ROC analysis, hierarchical logistic regression, and reclassification metrics. Nonlinear relationships were explored using restricted cubic spline analysis, and clinical utility was assessed by decision curve analysis. *Results:* PDW was significantly correlated with residual SYNTAX score (Spearman ρ = 0.503, *p* < 0.001) and increased progressively across SYNTAX severity strata and clinical presentation groups. In multivariable analysis, PDW remained independently associated with high residual SYNTAX score (OR 1.38, 95% CI 1.07–1.82, *p* = 0.016). The addition of PDW to a hierarchical clinical model significantly improved model performance (ΔR^2^ = 0.049, *p* = 0.012). Although the improvement in area under the curve (AUC) was modest, reclassification analyses demonstrated significant net reclassification and discrimination improvements. Spline analysis revealed a nonlinear relationship, with a marked increase in risk beyond PDW levels of approximately 13 fL. Decision curve analysis confirmed the clinical utility of PDW across a range of threshold probabilities. *Conclusions*: PDW is independently associated with post-procedural coronary anatomical complexity and provides incremental predictive value beyond established clinical variables. However, PDW should be interpreted as a biomarker reflecting platelet heterogeneity within a thromboinflammatory context, without the ability to distinguish between acute and chronic components.

## 1. Introduction

Multivessel coronary artery disease (CAD) is the dominant angiographic pattern encountered among patients presenting with both acute coronary syndromes (ACSs) and chronic coronary syndrome (CCS), affecting a substantial proportion of individuals referred for invasive coronary evaluation [1,2]. In this population, the extent and complexity of anatomical disease remaining after the index percutaneous coronary intervention (PCI), expressed as the post-procedural SYNTAX (Synergy between Percutaneous Coronary Intervention with Taxus and Cardiac Surgery) score, has emerged as a powerful determinant of subsequent adverse cardiovascular events, independent of the presenting clinical syndrome [3,4,5]. Several studies and registry analyses have consistently shown that a high post-PCI SYNTAX score, reflecting incomplete anatomical revascularization, is associated with elevated long-term mortality, recurrent myocardial infarction, and the need for repeat revascularization procedures [3,6,7].

Beyond anatomical and procedural factors, cardiovascular risk is increasingly recognized as a multidimensional construct influenced by biological, behavioral, and social determinants, underscoring the need for comprehensive risk assessment strategies that integrate multiple domains [8].

Current guidelines for ST-elevation myocardial infarction (STEMI) and non-ST-elevation myocardial infarction (NSTEMI) preferentially endorse a culprit-lesion-only PCI strategy during the index procedure, reserving non-culprit revascularization for a staged elective setting [1]. While this approach reduces the procedural risk of the acute phase, it inevitably leaves a measurable untreated disease burden in patients with multivessel involvement. Given the prognostic weight of this remaining disease burden, accurate estimation of post-PCI anatomical complexity at the time of the index procedure, before staging decisions are finalised, would be of considerable clinical value. Yet the SYNTAX score calculation demands meticulous angiographic analysis and is not readily obtainable outside of catheterization laboratories; a routinely available laboratory surrogate capable of reflecting overall coronary disease load would therefore be highly desirable.

Platelets occupy a central position in the pathophysiology of coronary atherosclerosis that extends well beyond their canonical role in acute thrombosis. Experimental and clinical evidence accumulated over recent years has established that platelet activation contributes to atherosclerotic plaque initiation, progression, and destabilization through direct cell-to-cell interactions, the release of pro-inflammatory mediators, and the promotion of a pro-coagulant vascular microenvironment [9,10]. In particular, newly released, metabolically active platelets, termed reticulated or immature platelets, exhibit heightened reactivity, increased thromboxane A_2_ synthesis, and augmented surface expression of glycoprotein receptors compared with older circulating platelets [9,10]. The abundance of these functionally distinct platelet subpopulations rises under conditions of accelerated megakaryopoiesis, which is itself upregulated by myocardial ischemia, plaque-mediated thrombin generation, and systemic inflammatory signaling [10].

PDW is a standard component of the automated complete blood count that quantifies the degree of variation in platelet size across the circulating population. As a morphometric index, PDW reflects the heterogeneity of the platelet pool and correlates with the proportion of large, metabolically immature reticulated platelets, thereby serving as an indirect proxy for ongoing platelet turnover and heightened platelet reactivity [9]. Unlike mean platelet volume (MPV), which captures only the average size, PDW conveys information about the breadth of the size distribution and may therefore be a more sensitive indicator of shifts in platelet population dynamics under thrombotic stress [11,12]. Importantly, PDW can be extracted from routine blood count results at no additional cost, making it an immediately accessible and universally available biomarker in virtually any clinical setting.

Several investigations have linked elevated PDW to adverse outcomes in the setting of ACS. Increased PDW has been associated with greater infarct severity, reduced thrombolysis success in STEMI, higher SYNTAX scores at presentation, long-term major adverse cardiovascular events (MACE) after NSTEMI, and unfavorable outcomes following coronary bifurcation PCI [13,14,15,16,17,18]. Despite this accumulating evidence, three important limitations characterize the existing literature. First, prior studies have predominantly examined the relationship between PDW and the total coronary disease burden at initial angiography, without specifically investigating whether PDW correlates with the anatomical complexity remaining after culprit-lesion PCI, a clinically distinct and arguably more actionable quantity. Second, the comparative performance of PDW against other hematologic indices, including red cell distribution width (RDW) and MPV, in predicting anatomical disease complexity has not been rigorously evaluated within a single cohort. Third, and perhaps most importantly, none of the prior studies have tested PDW’s incremental discriminative value over well-established clinical determinants of disease severity, most notably clinical presentation category, using formal hierarchical modelling.

The present study was therefore designed to address these gaps. In a retrospective cohort of patients with multivessel CAD managed with a culprit-lesion PCI strategy, we investigated: (1) whether PDW correlates with the post-PCI SYNTAX score as a continuous measure of coronary anatomical complexity; (2) whether PDW levels differ across clinical presentation categories (CCS, NSTEMI, and STEMI) and across SYNTAX score severity strata; (3) whether PDW is an independent predictor of high post-PCI SYNTAX score after adjustment for conventional cardiovascular risk factors and clinical presentation; and (4) whether the addition of PDW to a hierarchical prediction model provides significant incremental discriminative information beyond that afforded by clinical variables alone. To our knowledge, this represents the first study to examine the relationship between PDW and the post-procedural SYNTAX score in a multivessel CAD population encompassing the full spectrum of clinical presentation categories.

## 2. Materials and Methods

### 2.1. Study Design and Population

This retrospective observational study was conducted at Gaziantep University Şahinbey Research and Application Hospital. The study population consisted of patients who underwent percutaneous coronary intervention (PCI) for multivessel coronary artery disease between 1 January 2024 and 31 December 2025. Ethics committee approval was obtained prior to any data extraction or analysis (Gaziantep University Non-Interventional Clinical Research Ethics Committee; 4 February 2026; decision no: 2026/103). Due to the retrospective design and the use of anonymized hospital records, the requirement for individual informed consent was waived.

During this period, consecutive patients with angiographically confirmed multivessel coronary artery disease who underwent culprit-lesion-only PCI were identified from institutional electronic medical records and angiographic databases. After applying predefined inclusion and exclusion criteria, patients for whom a staged revascularization strategy was planned and who had complete clinical and laboratory data were included in the final analysis, resulting in a study population of 140 patients.

Multivessel coronary artery disease was defined as ≥50% stenosis in at least two major epicardial coronary arteries or involvement of the left main coronary artery. Patients with prior coronary artery bypass grafting, single-vessel disease, complete revascularization during the index procedure, active infection or inflammatory disease, hematological disorders affecting platelet indices, severe renal dysfunction (eGFR < 30 mL/min/1.73 m^2^), advanced liver disease, malignancy, or incomplete data were excluded.

A priori sample size estimation was performed during the ethics application based on previously reported associations between hematological indices and coronary artery disease complexity. Assuming a moderate effect size, a significance level of 0.05, and a statistical power of 80%, the minimum required sample size was estimated to be approximately 128 patients. The final study population of 140 patients satisfied these requirements.

### 2.2. Angiographic Analysis and Quantitative Coronary Angiography

Coronary angiography was performed using the standard Judkins technique with a Siemens Artis zee digital angiography system (Siemens Healthineers, Erlangen, Germany). Quantitative coronary angiography (QCA) measurements were obtained directly from the integrated digital angiographic workstation using dedicated software (syngo Dynamics QCA, Siemens Healthineers, Erlangen, Germany) on an Artis zee system (software version VD11C).

All angiographic and QCA analyses were independently performed by two experienced interventional cardiologists (each with >10 years of experience and >500 PCI procedures annually) who were blinded to clinical and laboratory data. In cases of disagreement, the angiographic images were re-evaluated by a third senior cardiologist, and a consensus decision was reached. The final SYNTAX score recorded in the database represented the consensus value; individual operator-level scores prior to adjudication were not systematically retained, precluding formal calculation of interobserver agreement statistics.

In addition to quantitative measurements, the anatomical complexity of coronary artery disease was evaluated using the post-procedural (residual) SYNTAX score based on visual assessment of angiographic images obtained after culprit-lesion PCI during the index procedure. This scoring system incorporates lesion characteristics such as lesion location, bifurcation involvement, total occlusion, lesion length, calcification, and vessel tortuosity to provide a comprehensive assessment of coronary disease burden. Quantitative coronary angiography measurements were used to support lesion assessment; however, SYNTAX score calculation was based on visual angiographic evaluation in accordance with standard methodology and did not directly rely on QCA-derived parameters.

Patients were stratified into two groups according to SYNTAX score as low (≤22) and high (≥23) SYNTAX groups. This cut-off was selected based on established SYNTAX score risk categories, in which scores ≤ 22 represent low anatomical complexity and scores ≥ 23 indicate intermediate-to-high complexity coronary artery disease, and was used as the primary framework for comparative analyses.

All angiographic and QCA analyses were independently performed by two experienced interventional cardiologists who were blinded to clinical and laboratory data. In cases of disagreement, the angiographic images were re-evaluated by a third senior cardiologist, and a consensus decision was reached.

### 2.3. Composite Index Calculations

To evaluate atherogenic and hematological burden, composite indices were calculated using standard formulas derived from laboratory measurements.

The atherogenic coefficient (AC) was calculated as follows:

AC = (Total cholesterol − HDL cholesterol)/HDL cholesterol.

This index reflects the relative proportion of non-high-density lipoprotein (HDL) cholesterol and provides an integrated measure of atherogenic lipid burden.

In addition, hematological indices including PDW, RDW, and MPV were evaluated as markers of platelet activation and hematological status.

### 2.4. Clinical and Laboratory Data

Clinical data including demographic characteristics, cardiovascular risk factors, and comorbid conditions were obtained from electronic medical records.

Venous blood samples were collected at the time of initial hospital admission, prior to coronary angiography and before any invasive procedure or pharmacological intervention, including antiplatelet loading doses. Therefore, PDW values reflect baseline platelet status before exposure to P2Y12 inhibitors administered during the index procedure.

Complete blood count parameters, including PDW, MPV, RDW, hemoglobin, and platelet count, were measured using an automated hematology analyzer (Sysmex XN-1000, Sysmex Corporation, Kobe, Japan) based on impedance and flow cytometry techniques. Biochemical parameters, including total cholesterol, HDL cholesterol, creatinine, AST, ALT, and high-sensitivity cardiac troponin, were measured using an automated chemistry analyzer (Roche Cobas 8000, Roche Diagnostics, Mannheim, Germany) using standardized enzymatic and immunoassay methods in accordance with manufacturer protocols.

### 2.5. Study Workflow

All patients underwent culprit-lesion PCI during the index procedure. In accordance with current clinical practice, a staged revascularization strategy was planned for remaining significant non-culprit lesions, and elective intervention was scheduled accordingly.

Residual SYNTAX score was calculated after the index procedure, reflecting the anatomical complexity of untreated coronary lesions. The relationship between admission laboratory parameters and residual coronary complexity was subsequently analyzed.

### 2.6. Ethics Statement

The study was approved by the Gaziantep University Non-Interventional Clinical Research Ethics Committee (approval date: 4 February 2026, decision number: 2026/103). Due to the retrospective design of the study and the use of anonymized patient data, the requirement for informed consent was waived. Patient inclusion was performed after ethics committee approval, and the study was conducted in accordance with the principles of the Declaration of Helsinki.

### 2.7. Statistical Analysis

All statistical analyses were performed using R statistical software (version 4.3; R Foundation for Statistical Computing, Vienna, Austria).

Continuous variables were tested for normality using the Shapiro–Wilk test and are presented as mean ± standard deviation or median [interquartile range], as appropriate. Categorical variables are expressed as counts and percentages.

Patients were stratified into two groups according to the residual SYNTAX score: low (≤22) and high (≥23). Between-group comparisons were performed using the independent-samples *t*-test or Mann–Whitney U test for continuous variables, as appropriate, and the chi-square test for categorical variables.

Patients were stratified into quartiles based on the sample distribution of PDW values using standard percentile-based definitions (Q1: ≤25th percentile; Q2: 25th–50th percentile; Q3: 50th–75th percentile; Q4: >75th percentile). These quartile boundaries were determined independently of and prior to any outcome-based analyses. Comparisons across PDW quartiles were performed using the Kruskal–Wallis test, followed by Bonferroni-adjusted post hoc pairwise comparisons where applicable.

The association between PDW and residual SYNTAX score was assessed using Spearman rank correlation analysis. To evaluate whether this association was independent of clinical presentation, partial Spearman correlation analysis was performed using a residual-based approach. Formal mediation analysis was conducted using the Baron and Kenny framework to decompose the total effect into direct and indirect (mediated) components; bootstrap resampling (1000 iterations) was used to derive 95% confidence intervals for the indirect effect. Interaction between PDW and clinical presentation was tested using a linear regression model including mean-centered PDW, mean-centered clinical presentation, and their product term. Stratified correlation analyses were performed within each clinical presentation category. These analyses are presented in Supplementary Table S1.

Univariate and multivariate logistic regression analyses were performed to identify predictors of high residual SYNTAX score (≥23). Results are reported as odds ratios (ORs) with 95% confidence intervals (CIs). Multicollinearity was assessed using the variance inflation factor (Supplementary Table S3).

Model discrimination was evaluated using receiver operating characteristic (ROC) curve analysis, and the area under the curve (AUC) was calculated. The optimal PDW cut-off value was determined using Youden’s index. Comparisons between ROC curves were performed using DeLong’s test (Supplementary Table S4).

To assess incremental predictive value, hierarchical logistic regression models were constructed by sequentially adding variable blocks (M1–M5). Model performance was evaluated using Nagelkerke pseudo-R^2^ and likelihood ratio tests.

Reclassification performance was further evaluated using net reclassification improvement (NRI) and integrated discrimination improvement (IDI) (Supplementary Table S5).

Nonlinear associations between PDW and high residual SYNTAX score were assessed using restricted cubic spline analysis.

Decision curve analysis (DCA) was performed to evaluate the clinical utility of the PDW-inclusive model.

Sensitivity analyses, including outlier exclusion using Cook’s distance, and sample size adequacy assessments were performed and are provided in Supplementary Tables S6 and S7.

A two-sided *p*-value < 0.05 was considered statistically significant.

## 3. Results

### 3.1. Study Population and Baseline Characteristics

A total of 140 patients with multivessel coronary artery disease undergoing coronary angiography and staged revascularization were included. The mean age was 62.9 ± 10.0 years, and 89 patients (63.6%) were male. Diabetes mellitus and hypertension were present in 67 (47.9%) and 95 (67.9%) patients, respectively.

NSTEMI was the most frequent clinical presentation (45.7%), followed by STEMI (30.7%) and CCS (23.6%). The LAD was the most common culprit vessel (*n* = 55, 39.3%), followed by CX (*n* = 46, 32.9%) and RCA (*n* = 39, 27.9%). The most frequent non-culprit target vessel was the RCA (*n* = 51, 36.4%), followed by LAD (*n* = 47, 33.6%) and CX (*n* = 42, 30.0%). The median time from index PCI to planned elective revascularization was 20 (11–32) days.

Patients were stratified into low (≤22, *n* = 89) and high (≥23, *n* = 51) residual SYNTAX groups. Baseline demographic and clinical characteristics were comparable between groups (Table 1). However, clinical presentation differed significantly (*p* < 0.001), with STEMI more frequent in the high SYNTAX group, whereas CCS predominated in the low SYNTAX group.

**Figure 1:**
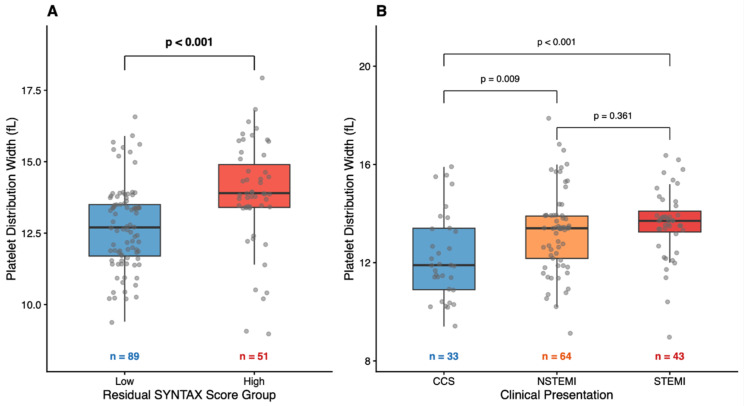
Platelet distribution width (PDW) according to (**A**) residual SYNTAX score group and (**B**) clinical presentation. (**A**) PDW values were significantly higher in patients with high residual SYNTAX score (≥23) compared with low SYNTAX score (≤22). (**B**) PDW progressively increased from CCS to NSTEMI and STEMI. Data are presented as median with interquartile range; dots represent individual observations. Statistical comparisons were performed using the Mann–Whitney U test (**A**) and Kruskal–Wallis test with Bonferroni post hoc correction (**B**).

Among laboratory parameters, PDW was significantly higher in patients with high SYNTAX scores (13.9 [13.4–14.9] vs. 12.7 [11.7–13.5] fL, *p* < 0.001), while MPV, RDW, hemoglobin, lipid parameters, and renal function markers were similar between groups (all *p* > 0.05; Table 1). These findings are illustrated in Figure 1A. PDW also differed significantly across clinical presentation categories, with the highest values observed in STEMI patients (Figure 1B).

### 3.2. Correlation Between PDW and Residual SYNTAX Score

Spearman correlation analysis demonstrated that PDW was the only laboratory parameter significantly associated with residual SYNTAX score (ρ = 0.503, *p* < 0.001), whereas other hematologic and biochemical markers showed no significant correlations (Table 2).

**Figure 2:**
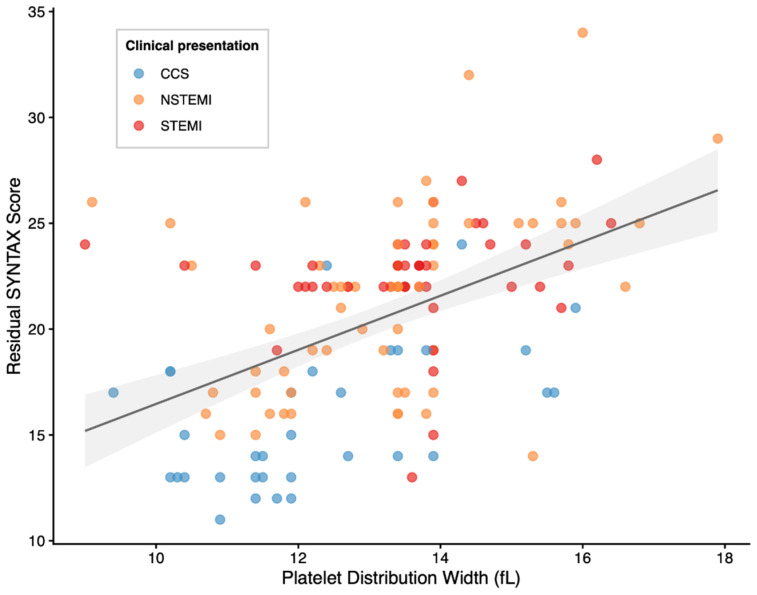
Scatter plot showing the relationship between PDW and residual SYNTAX score. A positive monotonic association was observed between PDW and SYNTAX score (Spearman ρ = 0.503, *p* < 0.001). The solid line represents the fitted trend with 95% confidence interval shading.

This relationship is visually supported by scatter analysis, demonstrating a positive monotonic association between PDW and SYNTAX score across clinical presentations (Figure 2).

#### Independence from Clinical Presentation

To assess whether the PDW–SYNTAX association was independent of clinical presentation, formal mediation and interaction analyses were performed (Supplementary Table S1). After adjustment for clinical presentation, the correlation between PDW and residual SYNTAX score remained highly significant (partial ρ = 0.455, *p* < 0.001), with only 9.6% attenuation from the crude estimate (ρ = 0.503). Formal mediation analysis using the Baron and Kenny framework demonstrated that the direct effect of PDW on high residual SYNTAX score remained statistically significant after accounting for the indirect pathway through clinical presentation (direct effect OR = 1.34, 95% CI 1.01–1.78, *p* = 0.040), with only 13.0% of the total effect mediated (bootstrap 95% CI for indirect effect: 0.05–0.42). A statistically significant interaction was observed between PDW and clinical presentation (interaction coefficient = −0.659, *p* = 0.030), indicating that the strength of the PDW–SYNTAX association differed across clinical subgroups. Stratified analyses revealed that the association was significant in CCS (ρ = 0.496, *p* = 0.003) and NSTEMI (ρ = 0.485, *p* < 0.001) but attenuated and non-significant in STEMI (ρ = 0.221, *p* = 0.154), likely reflecting reduced statistical power and restricted PDW variability in this subgroup. Collectively, these findings indicate that PDW provides independent biological information beyond clinical presentation.

### 3.3. Dose–Response Relationship Across PDW Quartiles

Patients were stratified into PDW quartiles, revealing a clear dose–response relationship between PDW and coronary anatomical complexity (Table 3).

**Figure 3:**
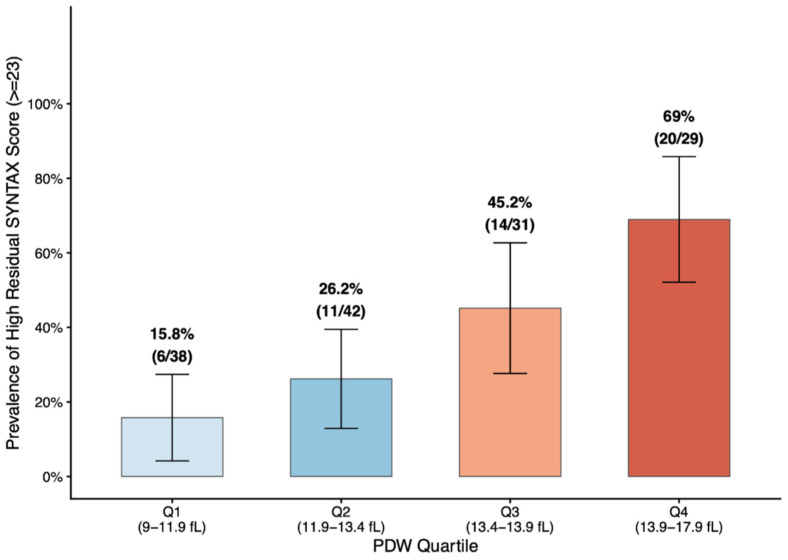
Prevalence of high residual SYNTAX score (≥23) across PDW quartiles.

Residual SYNTAX score increased progressively across quartiles (*p* < 0.001), and the prevalence of high SYNTAX score rose stepwise from 15.8% in Q1 to 69.0% in Q4 (*p* for trend < 0.001), as illustrated in Figure 3.

A stepwise increase in the proportion of high SYNTAX score was observed from the lowest to the highest PDW quartile, demonstrating a significant dose–response relationship (*p* for trend < 0.001).

NLR did not differ significantly across PDW quartiles (*p* = 0.380) and showed no significant trend (*p* for trend = 0.412). Importantly, other laboratory parameters such as RDW, MPV, and atherogenic coefficient did not show similar trends, underscoring the specificity of the PDW–SYNTAX relationship.

### 3.4. Predictors of High Residual SYNTAX Score

In univariate logistic regression analysis, PDW was significantly associated with high residual SYNTAX score (OR 1.54, 95% CI 1.21–1.96, *p* < 0.001). Clinical presentation was also a strong predictor, particularly NSTEMI and STEMI (Table 4).

The final multivariate model included 5 parameters with 51 events (high SYNTAX group), yielding an events-per-variable (EPV) ratio of 10.2, meeting the commonly recommended minimum threshold. The wide confidence intervals observed for clinical presentation (STEMI vs. CCS: OR 22.32, 95% CI 3.48–143.29) reflect near-complete separation between groups, with only 2 of 33 CCS patients (6.1%) classified as high SYNTAX compared with 23 of 43 STEMI patients (53.5%); these estimates should be interpreted with appropriate caution.

Using ROC analysis, PDW alone demonstrated an AUC of 0.722, with an optimal cut-off value of 13.4 fL determined by Youden’s J statistic, yielding a sensitivity of 80.4% and specificity of 59.6%. Notably, this ROC-derived threshold closely approximates the median PDW value (13.4 fL) and the inflection point identified in spline analysis (~13 fL); however, these values were derived through independent statistical procedures (percentile-based distribution, Youden’s index optimization, and nonlinear curve fitting, respectively), and their convergence reflects a consistent biological signal rather than circular optimization.

### 3.5. Incremental Predictive Value of PDW

Sequential modeling demonstrated that the addition of PDW significantly improved model performance. Incorporation of PDW (M5) resulted in a marked reduction in AIC (ΔAIC = −19.4) and a significant likelihood ratio improvement compared with the preceding model (*p* < 0.001; Table 5).

**Figure 4:**
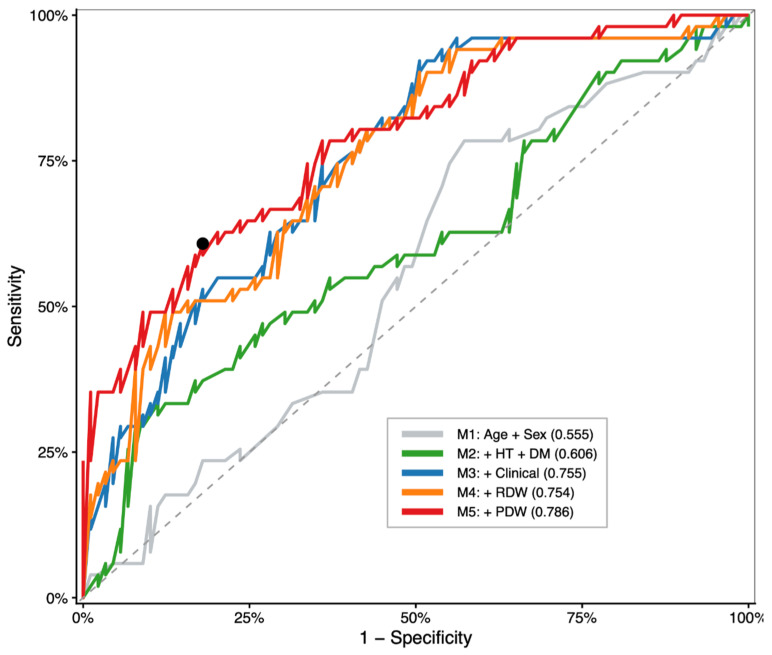
ROC curves for sequential predictive models.

ROC analysis showed progressive improvement across models, with the highest performance observed in the PDW-inclusive model (M5, AUC = 0.786) (Figure 4).

Model performance improved progressively with the addition of clinical and laboratory variables, with the highest discrimination observed in the PDW-inclusive model (M5). M1: age + sex; M2: M1 + diabetes + hypertension; M3: M2 + clinical presentation; M4: M3 + RDW; M5: M4 + PDW.

### 3.6. Nonlinear Association Between PDW and Coronary Complexity

**Figure 5:**
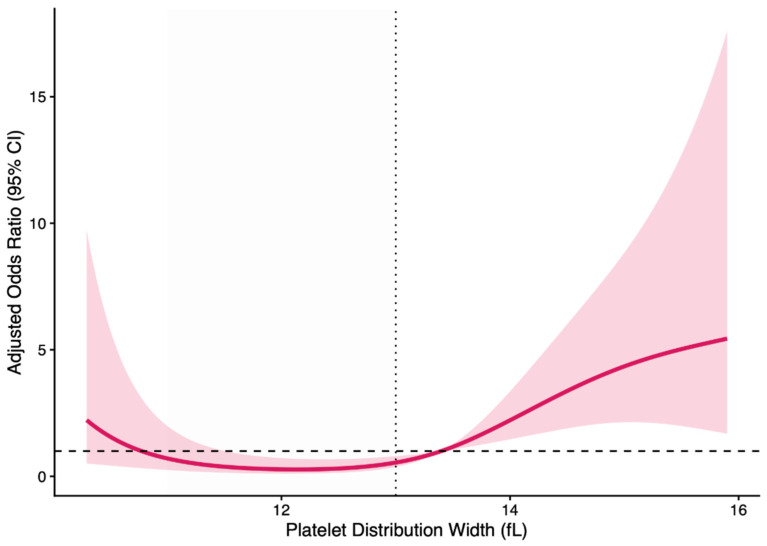
Restricted cubic spline analysis of the association between PDW and high residual SYNTAX score. A significant nonlinear relationship was observed (*p* for nonlinearity = 0.0026), with a threshold around 13 fL beyond which the risk increased steeply. The solid line represents adjusted odds ratios, and the shaded area indicates the 95% confidence interval.

Restricted cubic spline analysis demonstrated a significant nonlinear association between PDW and high residual SYNTAX score (*p* for nonlinearity = 0.0026). The risk remained relatively stable at lower PDW levels but increased sharply beyond approximately 13 fL, indicating a threshold-dependent relationship (Figure 5).

### 3.7. Clinical Utility

Decision curve analysis demonstrated that the PDW-inclusive model provided greater net clinical benefit compared with the clinical model alone across a wide range of threshold probabilities (Supplementary Figure S1).

### 3.8. Statistical Power

A priori power analysis indicated that a minimum sample size of 128 patients was required to detect a medium effect size (Cohen’s d = 0.50) with 80% power at a significance level of 0.05. The present study included 140 patients, exceeding this requirement.

## 4. Discussion

In this retrospective study of patients with multivessel CAD undergoing a culprit-lesion-only PCI strategy, we found that PDW is associated with post-procedural coronary anatomical complexity as reflected by the residual SYNTAX score. The main findings can be summarized as follows: PDW showed a positive association with residual SYNTAX score as a continuous indicator of disease burden; however, this relationship was most informative when interpreted in conjunction with clinical presentation. In multivariable analyses, PDW did not act as a dominant independent determinant but rather contributed modestly to the prediction of high residual SYNTAX score beyond conventional clinical variables. In addition, PDW levels increased stepwise across SYNTAX severity strata and clinical presentation categories, and incorporation of PDW into a hierarchical clinical model provided a significant incremental improvement in risk stratification.

The residual SYNTAX score is a well-established marker of incomplete revascularization and has consistently been linked to adverse cardiovascular outcomes, including mortality and recurrent ischemic events [3,4,5,6,7]. In contemporary practice—particularly in ACS settings—a culprit-lesion-only PCI strategy is frequently preferred, often leaving a considerable residual anatomical disease burden [1]. In this context, identifying simple and readily available biomarkers that can reflect residual coronary complexity remains clinically relevant.

Our results confirm that clinical presentation is the primary determinant of coronary anatomical complexity. Patients presenting with NSTEMI or STEMI had substantially higher residual SYNTAX scores, indicating a more diffuse and advanced disease phenotype. Within this framework, PDW should be interpreted as a complementary marker rather than a competing predictor. Notably, formal mediation and interaction analyses provided robust evidence that PDW contributes independent predictive information beyond clinical presentation. After adjustment for clinical presentation, the PDW–SYNTAX correlation was attenuated by only 9.6%, and mediation analysis using the Baron and Kenny framework demonstrated that only 13% of the total PDW effect was mediated through clinical presentation, with the direct effect remaining statistically significant (*p* = 0.040). Furthermore, a significant interaction between PDW and clinical presentation (*p* = 0.030) indicated effect modification, with the association being most pronounced in CCS and NSTEMI subgroups. The attenuated and non-significant correlation observed in the STEMI subgroup (ρ = 0.221, *p* = 0.154) likely reflects the smaller sample size and restricted PDW variability in this acute population rather than a true absence of biological association. These findings suggest that PDW is associated with coronary anatomical complexity within a thromboinflammatory context; however, they do not allow differentiation between acute and chronic biological contributions.

The observed relationship between PDW and coronary complexity is biologically plausible given the established role of platelets in atherosclerosis and thrombosis. However, the present data do not allow direct inference regarding specific biological mechanisms. Therefore, PDW should be interpreted as a clinical marker associated with coronary complexity rather than a mechanistic indicator of platelet turnover [9,10,18]. PDW reflects variability in platelet size distribution and has been associated with platelet heterogeneity in prior studies. However, the present data do not permit direct inference regarding specific platelet subpopulations or underlying biological mechanisms [9,10]. Therefore, elevated PDW may represent an integrated marker of increased platelet turnover and systemic thromboinflammatory activation. This phenotype may contribute to more diffuse coronary involvement, providing a mechanistic link between PDW and residual disease burden.

It should be noted that the mediation analysis was performed using the Baron and Kenny framework, which has known methodological limitations, including assumptions of linearity and susceptibility to unmeasured confounding between the mediator and outcome. Therefore, these findings should be considered exploratory and hypothesis generating rather than definitive evidence of causal pathways.

Previous studies have reported associations between PDW and the extent of coronary artery disease or adverse outcomes in ACS populations [14,15,16,17,18,19]. However, these studies largely focused on baseline angiographic findings or clinical endpoints. Our study extends the existing literature by specifically examining post-PCI residual anatomical complexity, which is more directly relevant to subsequent management decisions and prognosis.

An important observation in our cohort was that PDW showed a more consistent association with residual SYNTAX score compared to other hematological indices. Despite their physiological relationship, MPV was not significantly associated with residual SYNTAX score. Although prior studies have linked PDW with reticulated platelet fractions, the present study does not include direct measures of platelet subpopulations; therefore, such interpretations remain speculative [11,12].

A notable strength of this study is the evaluation of incremental predictive value using hierarchical modeling. Although clinical presentation remained the dominant determinant, adding PDW resulted in a statistically significant improvement in model performance (ΔR^2^ = 0.049, *p* = 0.012). While the increase in AUC was not statistically significant, this is not unexpected, as AUC is known to be relatively insensitive to incremental improvements when strong baseline predictors are present [20]. In this setting, improvements in reclassification metrics (NRI and IDI) and decision curve analysis provide more meaningful evidence of clinical utility.

An important consideration is whether admission PDW in ACS patients reflects the acute thromboinflammatory state triggered by plaque rupture or underlying platelet biology associated with chronic coronary disease. In ACS, platelet activation is rapidly induced by plaque rupture and amplified by inflammatory and neurohumoral pathways. Consequently, PDW measured at admission may be influenced by the acute phase of the index event. Given the cross-sectional design and single time-point measurement, the present study cannot distinguish between acute and chronic contributors to PDW elevation.

From a mechanistic perspective, PDW reflects variability in platelet size distribution and is associated with heterogeneity in circulating platelet populations. Such heterogeneity may arise from both acute thromboinflammatory activation and longer-term alterations in platelet turnover. However, given that PDW was measured at a single time point at admission, the relative contribution of these mechanisms cannot be determined in the present study. Therefore, PDW should be interpreted as a composite marker within a thromboinflammatory context rather than a specific indicator of chronic platelet activation.

PDW should therefore be interpreted as a risk-modifying marker rather than a primary determinant of coronary complexity. It appears to act as a biological amplifier, reflecting thromboinflammatory activity not fully captured by conventional clinical variables.

The nonlinear relationship between PDW and high residual SYNTAX score provides additional insight. Restricted cubic spline analysis suggested a relatively stable risk at lower PDW levels, followed by a progressive increase at higher values. Although this pattern may indicate a threshold effect, it should be interpreted cautiously, as it is data-driven and not externally validated. Rather than defining a strict cut-off, these findings suggest that higher PDW levels are associated with a disproportionate increase in coronary complexity.

From a practical standpoint, PDW has several advantages that support its potential role in early risk stratification. It is inexpensive, universally available as part of the routine complete blood count, and rapidly obtainable at the time of admission—before angiographic assessment is performed. In patients presenting with ACS and multivessel disease, elevated PDW at admission may serve as an early signal of greater residual coronary complexity, potentially informing decisions regarding the timing and completeness of staged revascularization. While PDW cannot replace comprehensive angiographic assessment, it may help identify patients who warrant more aggressive diagnostic evaluation or closer follow-up. This is further supported by decision curve analysis, which demonstrated improved net benefit across a wide range of threshold probabilities when PDW was included in predictive models. Importantly, the incremental value of PDW was most evident in reclassification analyses, suggesting that it may meaningfully improve individual patient risk classification even when global discrimination metrics show modest improvement. Although NLR was available, it was not significantly associated with residual SYNTAX score and was not retained in the final multivariable model. Importantly, other key inflammatory biomarkers such as C-reactive protein (CRP) and interleukin-6 (IL-6) were not available. The absence of these markers limits comprehensive assessment of systemic inflammation and raises the possibility of residual confounding in the observed PDW–SYNTAX relationship.

Overall, PDW emerges as a clinically relevant adjunctive biomarker that refines risk stratification within an established clinical framework rather than acting as a standalone predictor.

Taken together, these findings position PDW as a clinically relevant adjunctive biomarker that enhances risk stratification rather than acting as a standalone predictor of coronary complexity.

## 5. Study Limitations

Several limitations of the present study should be acknowledged.

First, the retrospective, single-center design introduces the possibility of selection bias and limits generalizability. Although consecutive patients were included and strict criteria were applied, external validation in larger, multicenter cohorts is needed.

Second, while the overall sample size met a priori power requirements and the final multivariate model achieved an acceptable events-per-variable ratio (EPV = 10.2 with 51 events and 5 parameters), the study remains relatively modest for subgroup analyses. The full model evaluated during backward selection included up to 14 candidate variables (EPV = 3.6), which is below recommended thresholds; however, the stepwise elimination procedure was specifically employed to reduce model complexity and mitigate overfitting risk. The wide confidence intervals observed for clinical presentation variables (e.g., STEMI vs. CCS: OR 22.32, 95% CI 3.48–143.29) reflect near-complete separation between groups and should be interpreted with caution. Similarly, subgroup analyses by PDW quartiles (Q1: *n* = 38, Q2: *n* = 42, Q3: *n* = 31, Q4: *n* = 29) and clinical presentation (CCS: *n* = 33, STEMI: *n* = 43) are exploratory and may be underpowered to detect smaller effect sizes.

Third, residual confounding cannot be entirely excluded despite multivariable adjustment and sensitivity analyses. Although NLR was included as a marker of systemic inflammation, other established inflammatory biomarkers such as CRP and IL-6 were not available, and therefore inflammatory pathways may not have been fully captured.

In addition, although blood samples were obtained prior to antiplatelet loading during the index procedure, information on chronic antiplatelet therapy before admission was not systematically recorded. Differences in pre-existing antiplatelet use between CCS and ACS populations may therefore represent an additional source of unmeasured confounding. Fourth, PDW was measured only at admission, prior to PCI. In ACS patients—particularly STEMI—platelet activation begins at the time of plaque rupture and is further amplified by the inflammatory cascade and catecholamine surge. Consequently, admission PDW may partially reflect the acute thromboinflammatory state rather than chronic platelet biology alone, introducing potential circular inference.

Given the cross-sectional design and single time-point measurement, the study cannot determine whether PDW reflects baseline platelet characteristics or acute changes related to the index event. This limitation is particularly relevant in ACS populations, where platelet activation is dynamically influenced by the acute thromboinflammatory milieu.

Fifth, although the residual SYNTAX score is well validated, it is based on angiographic assessment and subject to interobserver variability. This was addressed by independent evaluation by experienced interventional cardiologists blinded to clinical data, with consensus adjudication when necessary.

Sixth, formal interobserver agreement statistics (intraclass correlation coefficient or weighted kappa) for SYNTAX score assessment were not calculated, as individual operator-level scores were not systematically recorded prior to consensus adjudication. Although our protocol employed blinded independent evaluation by two experienced operators with senior adjudication for discrepancies—consistent with methodologies used in major multicenter studies—the lack of quantified reproducibility metrics represents a methodological limitation. However, the use of a binary SYNTAX classification (≤22 vs. ≥23) reduces the impact of minor scoring variations on outcome classification, and published validation studies have demonstrated acceptable interobserver reproducibility (ICC 0.82–0.89) for SYNTAX scoring by experienced operators.

Seventh, patients with severe renal dysfunction (eGFR < 30 mL/min/1.73 m^2^) were excluded to minimize confounding from uremic platelet dysfunction, which independently alters platelet morphology and size distribution. While this exclusion strengthens internal validity by isolating the PDW–coronary complexity relationship from uremia-induced platelet abnormalities, it limits the generalizability of our findings to patients with preserved or moderately reduced renal function.

Eighth, although PDW improved model performance, the magnitude of change in global discrimination metrics such as AUC was modest. However, this should be interpreted in light of the known limitations of AUC as a measure of incremental value. In contrast, reclassification metrics and decision curve analysis suggested meaningful improvements in patient-level risk assessment and potential clinical utility.

Finally, the observational design precludes any causal inference. The association between PDW and residual coronary complexity should therefore be interpreted as hypothesis-generating, and future prospective studies are needed to clarify whether PDW-guided strategies can improve clinical decision-making and outcomes.

Despite these limitations, the present study provides a comprehensive and methodologically robust evaluation of the relationship between PDW and post-procedural coronary complexity, integrating correlation, regression, nonlinear modeling, and clinical utility analyses within a single cohort.

## 6. Conclusions

In patients with multivessel CAD undergoing a culprit-lesion-only PCI strategy, PDW is independently associated with post-procedural coronary anatomical complexity. While the improvement in global discrimination metrics (AUC) was modest, PDW provided statistically significant incremental predictive value as evidenced by improvements in model fit, reclassification indices (NRI and IDI), and net clinical benefit on decision curve analysis. PDW should be interpreted as an adjunctive biomarker reflecting integrated thromboinflammatory activity and should not be interpreted as a marker of chronic platelet activation given the cross-sectional design and single time-point measurement.

## Figures and Tables

**Table 1:** Baseline demographics, clinical characteristics, and laboratory parameters by residual SYNTAX score group.

Variable	Overall (*n* = 140)	Low SYNTAX (*n* = 89)	High SYNTAX (*n* = 51)	*p*
Baseline characteristics				
Age, years	63.0 [54.8–70.0]	63.0 [54.0–70.0]	63.0 [59.0–71.0]	0.283
Sex, *n* (%)				0.715
Male	89 (63.6)	58 (65.2)	31 (60.8)	
Female	51 (36.4)	31 (34.8)	20 (39.2)	
Diabetes mellitus, *n* (%)	67 (47.9)	45 (50.6)	22 (43.1)	0.482
Hypertension, *n* (%)	95 (67.9)	65 (73.0)	30 (58.8)	0.093
Clinical presentation, *n* (%)				<0.001
CCS	33 (23.6)	31 (34.8)	2 (3.9)	
NSTEMI	64 (45.7)	38 (42.7)	26 (51.0)	
STEMI	43 (30.7)	20 (22.5)	23 (45.1)	
Laboratory parameters				
PDW, fL	13.4 [11.9–13.9]	12.7 [11.7–13.5]	13.9 [13.4–14.9]	<0.001
MPV, fL	10.6 [10.1–11.2]	10.5 [10.1–11.2]	10.7 [10.1–11.1]	0.959
RDW, %	13.3 [12.7–14.1]	13.3 [12.7–14.0]	13.2 [12.9–14.4]	0.557
Neutrophil-to-lymphocyte ratio (NLR)	2.74 [1.81–3.62]	2.90 [1.88–3.79]	2.48 [1.68–3.05]	0.133
Hemoglobin, g/dL	13.8 ± 1.8	14.0 ± 1.8	13.5 ± 2.0	0.146
Platelet count, ×10^3^/µL	262.0 [210.8–315.2]	263.0 [211.0–315.0]	251.0 [211.0–312.5]	0.976
Atherogenic coefficient	3.7 [3.2–4.3]	3.7 [3.1–4.2]	3.7 [3.3–4.6]	0.433
Creatinine, mg/dL	0.9 [0.7–1.0]	0.9 [0.7–1.0]	0.9 [0.7–1.0]	0.907
eGFR, mL/min/1.73 m^2^	85.5 ± 17.7	85.2 ± 18.1	86.0 ± 17.2	0.803
Albumin, g/L	39.8 ± 4.9	39.3 ± 5.2	40.6 ± 4.3	0.133
Antiplatelet treatment				
Clopidogrel, *n* (%)	68 (48.6)	47 (52.8)	21 (41.2)	0.220
Ticagrelor, *n* (%)	60 (42.9)	33 (37.1)	27 (52.9)	0.078
Prasugrel, *n* (%)	12 (8.6)	9 (10.1)	3 (5.9)	0.536

Data are presented as mean ± SD, median [interquartile range], or *n* (%). Low SYNTAX: residual SYNTAX score ≤ 22; High SYNTAX: residual SYNTAX score ≥ 23. Continuous variables were compared using Student’s *t*-test or Mann–Whitney U test based on normality (Shapiro–Wilk). Categorical variables were compared using Chi-square or Fisher’s exact test. PDW, platelet distribution width; MPV, mean platelet volume; RDW, red cell distribution width; eGFR, estimated glomerular filtration rate; AC, atherogenic coefficient; CCS, chronic coronary syndrome; NSTEMI, non-ST-elevation myocardial infarction; STEMI, ST-elevation myocardial infarction; NLR, Neutrophil-to-lymphocyte ratio. Antiplatelet treatment data refer to agents administered during or after the index PCI procedure; blood samples for PDW measurement were obtained prior to antiplatelet loading.

**Table 2:** Correlation between PDW and laboratory parameters with residual SYNTAX score.

Variable	Spearman *ρ*	*p* Value
PDW, fL	0.503	**<0.001**
Neutrophil-to-lymphocyte ratio (NLR)	−0.104	0.222
hs-Troponin, ng/L	0.114	0.179
MPV, fL	−0.030	0.721
Hemoglobin, g/dL	−0.106	0.210
RDW, %	0.054	0.523
Atherogenic coefficient	0.053	0.532

ρ, Spearman rank correlation coefficient; PDW, platelet distribution width; MPV, mean platelet volume; RDW, red cell distribution width; AC, atherogenic coefficient; NLR, Neutrophil-to-lymphocyte ratio. Bold *p* values indicate statistical significance (*p* < 0.05).

**Table 3:** Baseline, angiographic, and laboratory characteristics according to PDW quartiles.

	Q1 (*n* = 38)	Q2 (*n* = 42)	Q3 (*n* = 31)	Q4 (*n* = 29)	*p*	*p* Trend
PDW range, fL	9.0–11.9	12.0–13.4	13.5–13.9	14.3–17.9		
Baseline characteristics						
Age, years	63.5 [56.0–68.8]	65.5 [53.0–73.8]	62.0 [54.0–68.5]	63.0 [58.0–68.0]	0.795	0.878
Male, *n* (%)	22 (57.9)	27 (64.3)	21 (67.7)	19 (65.5)	0.845	0.461
Diabetes mellitus, *n* (%)	20 (52.6)	17 (40.5)	17 (54.8)	13 (44.8)	0.577	0.827
Hypertension, *n* (%)	27 (71.1)	29 (69.0)	19 (61.3)	20 (69.0)	0.841	0.665
Clinical presentation, *n* (%)					<0.001	
CCS	19 (50.0)	7 (16.7)	2 (6.5)	5 (17.2)		
NSTEMI	15 (39.5)	24 (57.1)	12 (38.7)	13 (44.8)		
STEMI	4 (10.5)	11 (26.2)	17 (54.8)	11 (37.9)		
Angiographic parameters						
Residual SYNTAX score	16.0 [13.0–18.0]	22.0 [19.0–22.8]	22.0 [19.0–23.5]	25.0 [22.0–25.0]	<0.001	<0.001
High SYNTAX, *n* (%)	6 (15.8)	11 (26.2)	14 (45.2)	20 (69.0)	<0.001	<0.001
Laboratory parameters						
MPV, fL	10.4 ± 0.8	10.5 ± 0.7	10.9 ± 0.8	11.0 ± 1.0	0.006	<0.001
RDW, %	13.2 [12.9–13.9]	13.4 [12.6–14.6]	13.3 [13.0–14.0]	13.2 [12.6–13.7]	0.662	0.937
Neutrophil-to-lymphocyte ratio (NLR)	2.95 [1.98–4.11]	2.73 [1.84–3.77]	2.62 [1.70–3.34]	2.51 [1.63–3.08]	0.380	0.412
Hemoglobin, g/dL	13.7 ± 1.7	13.9 ± 1.8	13.7 ± 2.0	13.8 ± 1.9	0.960	0.965
Atherogenic coefficient	3.7 [3.4–4.1]	3.7 [3.2–4.5]	3.8 [2.8–4.3]	3.5 [3.2–4.5]	0.972	0.716
Creatinine, mg/dL	0.9 [0.8–1.1]	0.9 [0.8–0.9]	0.9 [0.7–1.1]	0.9 [0.8–1.0]	0.368	0.185
eGFR, mL/min/1.73 m^2^	80.4 ± 17.0	89.5 ± 16.5	84.5 ± 19.6	87.3 ± 17.4	0.128	0.207
hs-Troponin, ng/L	9.1 [4.5–37.8]	9.4 [5.0–16.8]	12.3 [6.3–557.5]	9.4 [5.9–31.7]	0.392	0.473

Data are presented as mean ± SD, median [interquartile range], or n (%). *p* values from ANOVA or Kruskal–Wallis test for continuous variables and Chi-square or Fisher’s exact test for categorical variables. *p* trend from Jonckheere-Terpstra test (continuous) or Cochran-Armitage test (categorical). Bold *p* values indicate statistical significance (*p* < 0.05). PDW, platelet distribution width; MPV, mean platelet volume; RDW, red cell distribution width; eGFR, estimated glomerular filtration rate; AC, atherogenic coefficient; CCS, chronic coronary syndrome; NSTEMI, non-ST-elevation myocardial infarction; STEMI, ST-elevation myocardial infarction; NLR, Neutrophil-to-lymphocyte ratio; High SYNTAX, residual SYNTAX score ≥ 23. Quartile boundaries were defined using standard percentile-based cut-points derived from the sample distribution of PDW values, independent of outcome-based analyses.

**Table 4:** Univariate and multivariate logistic regression analysis for predictors of high residual SYNTAX score (≥23).

	Univariate		Multivariate	
Variable	OR (95% CI)	*p*	OR (95% CI)	*p*
Age, years	1.021 (0.986–1.057)	0.249	—	—
Male sex	0.828 (0.407–1.686)	0.604	—	—
Diabetes mellitus	0.742 (0.371–1.483)	0.398	—	—
Hypertension	0.527 (0.255–1.092)	0.085	0.458 (0.181–1.156)	0.098
NSTEMI vs. CCS	10.605 (2.332–48.220)	**0.002**	12.198 (1.980–75.131)	**0.007**
STEMI vs. CCS	17.825 (3.782–84.015)	**<0.001**	22.318 (3.476–143.289)	**0.001**
PDW, fL	1.541 (1.214–1.957)	**<0.001**	1.342 (1.013–1.778)	**0.040**
MPV, fL	0.925 (0.613–1.397)	0.712	—	—
RDW, %	1.145 (0.883–1.483)	0.307	—	—
NLR (per unit increase)	0.84 (0.65–1.00)	0.133	—	—
LDL cholesterol, mg/dL	1.013 (1.001–1.026)	0.033	—	—
ALT, U/L	1.022 (0.994–1.050)	0.131	—	—
hs-Troponin, per 100 ng/L	1.012 (0.999–1.024)	0.074	—	—
Atherogenic coefficient	1.140 (0.869–1.497)	0.343	—	—
Hemoglobin, g/dL	0.867 (0.716–1.051)	0.146	0.827 (0.654–1.044)	0.111
eGFR, mL/min/1.73 m^2^	1.003 (0.983–1.022)	0.801	—	—

OR, odds ratio; CI, confidence interval; CCS, chronic coronary syndrome; NSTEMI, non-ST-elevation myocardial infarction; STEMI, ST-elevation myocardial infarction; PDW, platelet distribution width; MPV, mean platelet volume; RDW, red cell distribution width; eGFR, estimated glomerular filtration rate; ALT, alanine aminotransferase; hs-Troponin, high-sensitivity troponin; NLR, Neutrophil-to-lymphocyte ratio. Univariate analysis was performed for all candidate predictors. Multivariate model was derived using backward stepwise elimination based on Akaike information criterion. LDL cholesterol, ALT, and hs-Troponin were included in the initial full model but were not retained in the final model. hs-Troponin OR is expressed per 100 ng/L increase due to the wide range of values. Bold *p* values indicate statistical significance (*p* < 0.05). —, not retained in the final multivariate model. The final multivariate model included 5 parameters with 51 events, yielding an events-per-variable (EPV) ratio of 10.2.

**Table 5:** Incremental predictive value of PDW for high residual SYNTAX score using sequential logistic regression models.

Model	Variables Included	Nagelkerke R^2^	ΔR^2^	*p* (Likelihood Ratio Test)
M1	Age, Sex	0.013	–	–
M2	M1 + Diabetes, Hypertension	0.052	+0.038	0.134
M3	M2 + Clinical presentation	0.252	+0.201	<0.001
M4	M3 + RDW	0.257	+0.004	0.463
M5	M4 + PDW	0.306	+0.049	0.012

Model definitions: M1, age + sex; M2, M1 + diabetes mellitus + hypertension; M3, M2 + clinical presentation (CCS, NSTEMI, STEMI); M4, M3 + red cell distribution width (RDW); M5, M4 + platelet distribution width (PDW). Abbreviations: PDW, platelet distribution width; RDW, red cell distribution width; CCS, chronic coronary syndrome; NSTEMI, non–ST-elevation myocardial infarction; STEMI, ST-elevation myocardial infarction. Model performance was evaluated using Nagelkerke pseudo-R^2^. ΔR^2^ represents the incremental increase compared with the preceding model. *p*-values were derived from likelihood ratio tests comparing nested models. Statistical significance was defined as *p* < 0.05.

## Data Availability

The datasets generated and/or analyzed during the current study are available from the corresponding author on reasonable request.

## Supplementary Materials

The following supporting information can be downloaded at https://www.mdpi.com/article/10.3390/medicina62050864/s1: Table S1. Assessment of confounding and interaction effects on the PDW–SYNTAX association. Table S2. Diagnostic performance of PDW for differentiating acute coronary syndrome from chronic coronary syndrome. Table S3. Assessment of multicollinearity among predictors using variance inflation factor (VIF). Table S4. Pairwise comparison of ROC curves using DeLong’s test across sequential prediction models. Table S5. Reclassification analysis: net reclassification improvement (NRI) and integrated discrimination improvement (IDI). Table S6. Sensitivity analysis: robustness of the PDW–SYNTAX association to outlier exclusion. Table S7. A priori power analysis. Figure S1. Decision curve analysis comparing clinical and PDW-inclusive models.

## Abbreviations

AC	atherogenic coefficient
ACS	acute coronary syndrome
AUC	area under the curve
CAD	coronary artery disease
CCS	chronic coronary syndrome
CI	confidence interval
eGFR	estimated glomerular filtration rate
HDL	high-density lipoprotein
IQR	interquartile range
MACE	major adverse cardiovascular events
MPV	mean platelet volume
NSTEMI	non-ST-elevation myocardial infarction
OR	odds ratio
PCI	percutaneous coronary intervention
PDW	platelet distribution width
QCA	quantitative coronary angiography
RDW	red cell distribution width
ROC	receiver operating characteristic
STEMI	ST-elevation myocardial infarction
SYNTAX	Synergy between Percutaneous Coronary Intervention with Taxus and Cardiac Surgery
VIF	variance inflation factor
