# Supplementary material for: Platelet Distribution Width Enhances Prediction of Residual Coronary Complexity Beyond Clinical Presentation in Patients Undergoing Culprit-Only PCI
Source: Medicina (Kaunas). 2026 Apr 30;62(5):864. doi: 10.3390/medicina62050864 (PMC13208271; doi:10.3390/medicina62050864)
Supplement: Supplementary file 1 [file medicina-62-00864-s001.zip › medicina-4242814-supplementary.pdf]

**Supplementary Table S1. Assessment of confounding and interaction effects on the PDW–SYNTAX association.**

| Analysis | Estimate | p value |
|---|---|---|
| Crude Spearman ρ | 0.503 | < 0.001 |
| Partial ρ (adjusted for clinical presentation) | 0.455 | < 0.001 |
| Attenuation (%) | 9.6 | — |
| Stratified: CCS (n = 33) | 0.496 | 0.003 |
| Stratified: NSTEMI (n = 64) | 0.485 | < 0.001 |
| Stratified: STEMI (n = 43) | 0.221 | 0.154 |
| Interaction term (PDW × Clinical presentation) | −0.659 | 0.030 |

*ρ, Spearman rank correlation coefficient; PDW, platelet distribution width; CCS, chronic coronary syndrome; NSTEMI, non-ST-elevation myocardial infarction; STEMI, ST-elevation myocardial infarction. Partial Spearman correlation was estimated using a residual-based approach. The interaction term was derived from a linear regression model including mean-centered PDW, mean-centered clinical presentation, and their product term. A significant interaction (p = 0.030) suggests that the strength of the PDW–SYNTAX association may differ across clinical subgroups. The non-significant STEMI result likely reflects the smaller sample size (n = 43) and restricted PDW range.*

**Supplementary Table S2. Diagnostic performance of PDW for differentiating acute coronary syndrome from chronic coronary syndrome.**

| Parameter | Value |
|---|---|
| AUC (95% CI) | 0.708 (0.597–0.819) |
| Optimal cut-off, fL | 11.95 |
| Sensitivity | 82.2% |
| Specificity | 57.6% |
| Positive predictive value | 86.3% |
| Negative predictive value | 50.0% |
| PDW — CCS, fL (median [IQR]) | 11.90 [10.90–13.40] |
| PDW — ACS, fL (median [IQR]) | 13.40 [12.40–13.90] |
| p value | < 0.001 |

*AUC, area under the receiver operating characteristic curve; CI, confidence interval; PDW, platelet distribution width; CCS, chronic coronary syndrome; ACS, acute coronary syndrome (NSTEMI + STEMI). Optimal cut-off was determined using Youden's J statistic. Group values are presented as median [interquartile range]. p value from Mann–Whitney U test.*

**Supplementary Table S3. Assessment of multicollinearity among predictors using variance inflation factor (VIF).**

| Variable | VIF |
|---|---|
| Age, years | 1.262 |
| Male sex | 1.471 |
| Diabetes mellitus | 1.139 |
| Hypertension | 1.156 |
| Clinical presentation | 1.320 |
| PDW, fL | 1.312 |
| MPV, fL | 1.232 |
| RDW, % | 1.213 |
| Atherogenic coefficient | 1.116 |
| eGFR, mL/min/1.73m² | 1.089 |
| Hemoglobin, g/dL | 1.623 |

*VIF, variance inflation factor; PDW, platelet distribution width; MPV, mean platelet volume; RDW, red cell distribution width; eGFR, estimated glomerular filtration rate. VIF values were calculated from the multivariable linear regression model. All VIF values were below 2.0, indicating no evidence of significant multicollinearity among predictors.*

**Supplementary Table S4. Pairwise comparison of ROC curves using DeLong's test across sequential prediction models.**

| Comparison | AUC (Model 1) | AUC (Model 2) | ΔAUC | Z statistic | p value |
|---|---|---|---|---|---|
| M1 vs M2 | 0.555 | 0.606 | 0.051 | −1.02 | 0.310 |
| M2 vs M3 | 0.606 | 0.755 | 0.149 | −3.65 | < 0.001 |
| M3 vs M4 | 0.755 | 0.754 | −0.001 | 0.04 | 0.966 |
| M4 vs M5 | 0.754 | 0.786 | 0.032 | −1.24 | 0.215 |
| M1 vs M5 | 0.555 | 0.786 | 0.231 | −4.01 | < 0.001 |

*AUC, area under the receiver operating characteristic curve; ΔAUC, difference in AUC between models. Model definitions: M1, age + sex; M2, M1 + hypertension + diabetes mellitus; M3, M2 + clinical presentation; M4, M3 + RDW; M5, M4 + PDW. Pairwise comparisons were performed using DeLong's method for correlated ROC curves. PDW, platelet distribution width; RDW, red cell distribution width. Bold p values indicate statistical significance (p < 0.05).*

**Supplementary Table S5. Reclassification analysis: net reclassification improvement (NRI) and integrated discrimination improvement (IDI).**

| Metric | Estimate | p value |
|---|---|---|
| Category-based NRI (95% CI) | 0.594 (0.281 to 0.829) | < 0.001 |
| NRI events | 0.191 | |
| NRI non-events | 0.403 | |
| IDI (95% CI) | 0.316 (0.179 to 0.480) | < 0.001 |
| ΔAUC (DeLong) | +0.032 | 0.215 |

*NRI, net reclassification improvement; IDI, integrated discrimination improvement; AUC, area under the curve; CI, confidence interval. Comparison: Model 4 (age + sex + HT + DM + clinical presentation + RDW) vs Model 5 (Model 4 + PDW). Category-based NRI was calculated using clinically relevant risk thresholds of 30% and 60%. Bootstrap resampling (B = 1,000; seed = 42) was used to derive 95% confidence intervals. Despite a non-significant change in AUC by DeLong's test, both NRI and IDI demonstrated highly significant reclassification improvement, indicating that PDW meaningfully improves risk stratification beyond conventional predictors.*

**Supplementary Table S6. Sensitivity analysis: robustness of the PDW–SYNTAX association to outlier exclusion.**

| Scenario | Spearman ρ | p value | AUC |
|---|---|---|---|
| Full dataset (N = 140) | 0.503 | < 0.001 | 0.722 |
| Excluding Cook's D > 4/N (N = 129) | 0.640 | < 0.001 | 0.808 |

*AUC, area under the receiver operating characteristic curve; PDW, platelet distribution width. Outlier detection was performed using Cook's distance (threshold 4/N = 0.029). Eleven influential observations were identified and excluded. Both the Spearman correlation and AUC strengthened after exclusion, confirming that the full-cohort estimates are conservative and not driven by outlier influence. AUC values correspond to the univariate discriminatory performance of PDW alone and are not directly comparable to the multivariable model-based AUC (0.786) reported in the main analysis.*

**Supplementary Table S7. A priori power analysis**

| Parameter | Value |
|---|---|
| Software | G*Power v3.1.9.7 |
| Analysis type | A priori power analysis |
| Statistical test | Independent-samples t-test (two-tailed) |
| Number of groups | 2 (Low SYNTAX vs High SYNTAX) |
| Effect size (Cohen's d) | 0.50 (medium effect) |
| Significance level (α) | 0.05 |
| Statistical power (1−β) | 0.80 |
| Total sample size required | 128 |
| Minimum per group | 64 |

*A priori power analysis was performed prior to data collection. A medium effect size (Cohen's d = 0.50) was assumed based on previously reported differences in hematological parameters between low and high SYNTAX score groups.*

**Supplementary Figure S1**. Decision curve analysis comparing clinical and PDW-inclusive models.

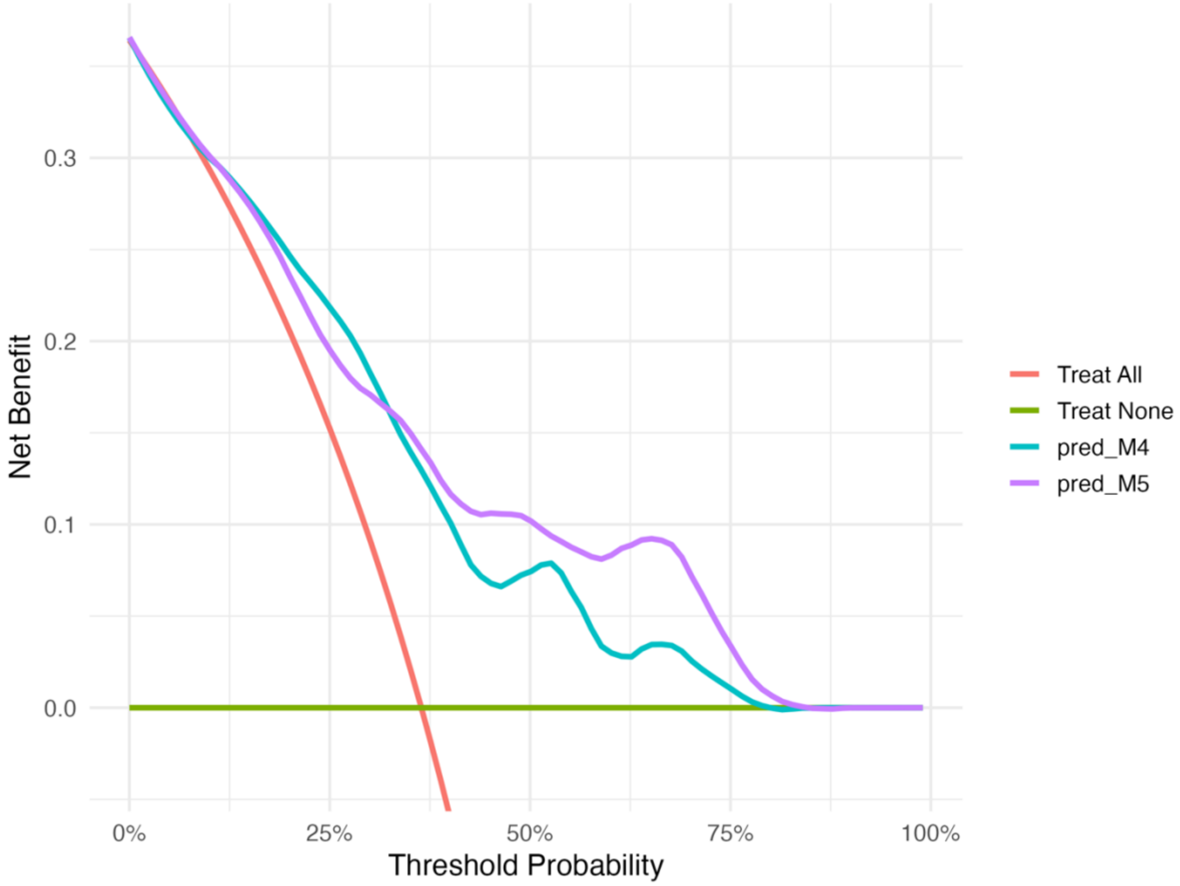


The PDW-inclusive model demonstrated greater net benefit across a wide range of threshold probabilities, indicating improved clinical utility compared with the clinical model alone.
